# The Precision Health and Everyday Democracy (PHED) Project: Protocol for a Transdisciplinary Collaboration on Health Equity and the Role of Health in Society

**DOI:** 10.2196/17324

**Published:** 2020-11-30

**Authors:** Michael Strange, Carol Nilsson, Slobodan Zdravkovic, Elisabeth Mangrio

**Affiliations:** 1 Department of Global Political Studies Malmö University Malmö Sweden; 2 Malmö Institute for Studies of Migration, Diversity & Welfare Malmö University Malmö Sweden; 3 Department of Experimental Medical Science Lund University Lund Sweden; 4 Department of Care Sciences Malmö University Malmö Sweden

**Keywords:** precision health, health care access, health literacy, everyday democracy

## Abstract

**Background:**

The project “Precision Health and Everyday Democracy” (PHED) is a transdisciplinary partnership that combines a diverse range of perspectives necessary for understanding the increasingly complex societal role played by modern health care and medical research. The term “precision health” is being increasingly used to express the need for greater awareness of environmental and genomic characteristics that may lead to divergent health outcomes between different groups within a population. Enhancing awareness of diversity has parallels with calls for “health democracy” and greater patient-public participation within health care and medical research. Approaching health care in this way goes beyond a narrow focus on the societal determinants of health, since it requires considering health as a deliberative space, which occurs often at the banal or everyday level. As an initial empirical focus, PHED is directed toward the health needs of marginalized migrants (including refugees and asylum seekers, as well as migrants with temporary residency, often involving a legally or economically precarious situation) as vulnerable groups that are often overlooked by health care. Developing new transdisciplinary knowledge on these groups provides the potential to enhance their wellbeing and benefit the wider society through challenging the exclusions of these groups that create pockets of extreme ill-health, which, as we see with COVID-19, should be better understood as “acts of self-harm” for the wider negative impact on humanity.

**Objective:**

We aim to establish and identify precision health strategies, as well as promote equal access to quality health care, drawing upon knowledge gained from studying the health care of marginalized migrants.

**Methods:**

The project is based in Sweden at Malmö and Lund Universities. At the outset, the network activities do not require ethical approval where they will not involve data collection, since the purpose of PHED is to strengthen international research contacts, establish new research within precision strategies, and construct educational research activities for junior colleagues within academia. However, whenever new research is funded and started, ethical approval for that specific data collection will be sought.

**Results:**

The PHED project has been funded from January 1, 2019. Results of the transdisciplinary collaboration will be disseminated via a series of international conferences, workshops, and web-based materials. To ensure the network project advances toward applied research, a major goal of dissemination is to produce tools for applied research, including information to enhance health accessibility for vulnerable communities, such as marginalized migrant populations in Sweden.

**Conclusions:**

There is a need to identify tools to enable the prevention and treatment of a wide spectrum of health-related outcomes and their link to social as well as environmental issues. There is also a need to identify and investigate barriers to precision health based on democratic principles.

**International Registered Report Identifier (IRRID):**

DERR1-10.2196/17324

## Introduction

Advances in medical research have led to an awareness that different populations respond differently to the same forms of treatment and are host to distinct pathogens [1]. In understanding how best to respond to these differences and the relative role played by genomic characteristics or environmental and lifestyle variables, there have been calls for so-called “precision health care” and “precision medicine.” The term “precision” denotes a sensitivity to these intergroup variables, pointing to the need to better model how environmental and genomic factors impact individuals unequally along socially and economically structured lines and with consequences for their health and receptivity to treatment. In this project, we refer to the health care, public health, and medical research aspects collectively as “precision health.”

According to Oh et al [1], the impact of precision health is of great importance because it is well known that generalizing results from research on one racial/ethnic group to another can work but may have fatal consequences when relevant differences, such as societal and genomic factors, are ignored.

The lack of diversity in large-scale biomedical studies hinders our understanding of human disease and severely limits our ability to develop optimal therapeutic interventions and treatments [1]. The basis of precision health is grounded in knowledge and effective communication between patients and health care professionals, including both those directly engaged in patient care and practitioners active in medical research. Communication within precision health care is important since social, environmental, and genetic factors have their roles as causes and explanations in order to be able to provide the right diagnoses and treatments at the right time [2]. Therefore, it is important to communicate with patients in order to identify those social and environmental factors that could be explanations.

Medical personnel need to make treatment decisions and predict efficacy of treatments to ensure health care is better suited to the diverse needs of different populations, as well as minimize negative side effects. Precision health requires the ability to identify subpopulations with susceptibilities to specific diseases, whether due to genetic or social determinants, and to understand how negative health care outcomes can develop through the complex environmental interactions that need to be understood through the social sciences. For example, environmental and lifestyle factors, such as housing challenges, economic stress, physical inactivity, and smoking, are quite frequently observed among marginalized migrants in Sweden [3,4], and this points to high public health inequality.

The position of being a migrant, unless well resourced, creates particular health consequences for individuals. Migration, particularly that which is involuntary or forced, is known to cause mental stress, and the circumstances surrounding the migration while escaping the native country are important [3]. The exodus from the country of origin may have been sudden, and if the reasons are war, disaster, and political persecution, there could be long stays in refugee camps. After arrival in the recipient country, there is usually a time of uncertainty during the asylum process, which might increase the risk of mental illness [5,6]. Furthermore, migrants have higher risks of depression, psychosis, and suicidal thoughts [5]. According to a study by Hjern [7], migrants of non-European background in Sweden are three to four times more likely to experience poor or very poor health when compared with Swedish-born individuals.

When it comes to cultural competence within health care, health care provision could be defined as a continuing process with the goal of effectively caring for people with culturally diverse backgrounds [8]. Having specific knowledge of health care professionals’ own cultural backgrounds, as well as patients’ cultural needs ensures holistic and competent care [8]. It is important to acknowledge where practitioners and patients have expertise in knowledge of different cultural practices, cultural assessments, and communication skills [8]. If patients and health care professionals do not fully understand each other, the relationship becomes undermined, leading to insufficient emotional support [9] and unmet health care needs, as they are not able to make themselves understood [10].

The need to improve the relationship between health care professionals and patients [1], as well as include more data that could help measure diverse needs within national populations [11], has parallels to debates over the applicability of democratic theories to the development of new participatory models for “good” health care systems [12-14], with more recent interest in bringing those same insights to medical research by, for example, involving patients in the stratification of research priorities [15-17].

## Methods

### Conceptual Background

The application of concepts first developed in the social sciences is fraught with difficulties given that the central term “democracy” is highly ambiguous and may lead to quite divergent outcomes, with mixed relevance to precision health. Enabling the patient’s ability to select from a menu of choices, such as the hospital or key aspects of their treatment, has been criticized by some for unfairly passing responsibility for complicated decisions to those lacking sufficient training, supporting a neoliberal marketization of health services [18,19]. Stark disparities in health literacy (ie, patients’ understanding of their health care needs and medical treatment) and access to medical services (eg, due to geographical proximity) are structural constraints not only limiting but also potentially opposing patients’ ability to choose. Such inequality, where particularly vulnerable groups are most often negatively affected, means the “patient-choice” model is easily co-opted by new public management models in which health care is a matter of private consumption rather than public good.

Nevertheless, the juxtaposition of “health” and “democracy” has introduced an important intervention by applying both normative and analytical categories developed around democratic theories within the social sciences to health care, a field with a very different ontology and series of practices. Doctor-patient relations have been traditionally hierarchical, and indeed good health care and medical research arguably rely upon a clear distinction between those roles with one party positioned as authoritative, as patients and medical practitioners rarely have equal access to medical expertise and practitioners’ authority has traditionally been a cornerstone of their professional legitimacy [20,21]. To speak of that relationship as “democratic” is potentially counter-intuitive. Despite this, several journals have emerged to focus on the application of democratic concepts to health care, and in several notable cases, such as the United Kingdom’s National Health Service [22], “democracy” has been adopted as the central principle of best practice in relations between health care practitioners and patients. “Health democracy” fosters both a fertile academic debate and policy developments.

Much of the policy discussion can, at least at first, be understood within a broader neoliberalization of health care based around the notion of new public management with patients as the “users” or “customers,” around which the economics of hospitals and medical research should orbit [23]. That connection may explain some of the initial drive for linking “democracy” to health care, by fostering a market model, and certainly much of the skepticism from those opposed to such a model [24]. That said, it is important to deconstruct the debate in order to identify those approaches that are of value. Since the late 1990s, there has been a paradigm shift within many Western countries, in which the authority of health care practitioners has been challenged by a series of “patient-centered” approaches [25-28]. There has been greater focus on designing health care provision as a series of choices from which the patient should choose, mirroring similar developments seen in the public sector within many Western states since the 1990s. Patient choice provided an important part of the mechanism through which this new form of accountancy was made possible, ensuring that there would be “consumers” able to redistribute finance along market principles. Choice-driven health care is based upon a simple (and not unproblematic) representative model of democracy, and the ability of patients to choose between multiple options of treatment is analogous to electoral rights [29].

However, where health democracy goes further than electoral democracy is its focus on the importance of informed consent, in which patients must be made aware with accessible information on their condition and the available options and likely outcomes [30]. Discussion has moved beyond analogies with electoral democracy to more substantive forms of democracy that acknowledge the need to not only ask the “public” but also facilitate dialogue that creates a subject able to act democratically, with awareness of their political being [31]. As such, in a critical response to the market-based approach, there has been growing attention to what can be learnt from deliberative models of democracy in the Habermasian tradition [30]. If “health democracy” is understood as following a deliberative model, a “good” health care system is one that incorporates whatever is needed to facilitate a dialogue in which patients and health care practitioners (eg, doctors) discuss openly such that each may understand the other’s perspective toward reaching a position where each feels having been heard [32].

The key intervention of a deliberative approach to democratic thought is that the individual who is to vote, for example, is treated not as someone already able to act democratically, but, rather, as someone who has to be made as such via a process that goes beyond the vote itself. For democracy to function effectively, there is a requirement of access to open media, as well as education, and other institutions necessary for maintaining the minimum engagement required for effective deliberation. For health democracy, this can be read in the same way, with a good health care system requiring not only that the patient’s views be heard, but also that the system be designed so as to ensure the patient can fulfill that role. This requires education and support for patients and their relatives to be “democratically” active, which are sensitive to the wider societal issues structuring their existing understanding of and access to health care. In recent years, this has included a new focus on posttreatment care to assist emotional and physical support, and counselling, as well as a better understand of the role of a patient’s family and social network in recovery [33,34]. Consequently, health democracy leads to a much more pervasive, complex, and richer model of health care in terms of practice, but with implications for medical research and public health reform [35].

Given, however, that health democracy requires a rethinking of how to not only design but also evaluate health care systems, what are the drivers behind its development? As mentioned earlier, a simple choice-based model of democracy fits health care after 1980, as new public management market-based models spread across the public sector. The rhetoric around patient choice, as well as closer direct involvement of patients in their health care, has featured prominently within political speeches and printed materials, suggesting that health democracy has a legitimating function within many contemporary societies [36]. Where patient groups have responded in turn by calling for enhanced patient choice, there is also a belief (whether true or not) that health democracy improves the quality of health care. It can be speculated that listening to patients’ diverse needs opens the health care system to a wider set of opinions and experiences among the studied group, making it easier to design optimal policies. Recent research on patient-public involvement within medical research indicates that there is much to be gained for all parties concerned if, for example, cancer patients and their families are able to meet with medical researchers and health policy makers to deliberate priorities [17,37]. These types of engagements are sometimes described as “coproduction,” a term developed within design studies and found increasingly in the social sciences [38]. In brief, coproduction requires engagement among private individuals, community associations, public service providers, and potentially private businesses for collaboratively deciding over funding priorities for usually public good. It is seen as a way to re-engage different actors within a broader maintenance of democratic society that counters a decline in public support for electoral democracy. Yet, beyond that normative goal, the project to combine a multiplicity of perspectives is driven also by a desire to promote environmental sustainability, viewing ecological damage as a product of exclusion where communities negatively affected by, for example, pollution are not able to influence decisions. Inclusion of those marginalized voices has the potential to not only aid those individuals [39], but also enhance the overall society by creating a check on unsustainable practices.

Consequently, health democracy places health care in its societal context, opening up the question of health care’s role in society. In addition to curing illness and enhancing health well-being, health care is one of the main ways in which individuals experience being part of an organized society. This is most acute in the case of marginalized communities whom may otherwise feel disenfranchised from the wider community. The “democratic” element of health care draws attention to the “everyday” experience of being in a society. First, health care and medical research are shaped by basic questions around inclusion and exclusion. There are several questions. Who is given what level of health care and which genomes and populations, as well as conditions, are considered within medical research? Moreover, how does population health vary across groups? Furthermore, who is given access to the resources necessary to be an informed patient? Second, a key challenge for deliberative debates is whether they can ever escape the power relations that structure how humans interact, whether understood along the lines of gender, race, and wealth, or other lines. Health care and medical research take place within power structures. Health democracy requires identifying those power relations. Finally, health democracy also points to the role of health care, public health, and medical research in empowering individuals to engage in society. It is well-established that in addiction and other lifestyle disorders, such as morbid obesity, patient self-empowerment greatly enhances the efficacy of treatment [40]. Democratic models that help better operationalize empowerment and how it might be best achieved have great potential to enhance health care.

Political science has traditionally studied democracy with respect to the decision-making institutions governing nation states, primarily focusing on electoral systems and the maintenance of civil rights. Health democracy’s turn toward questions of participation and deliberation, particularly in medical research, challenge an institutionalist model. For that reason, the project draws upon postinstitutionalist models that emphasize the importance of so-called “everyday” practices in which politics takes place. “Everyday” here is meant to mark out the banal largely ignored spheres of social life in which individuals interact and yet, as in the work of scholars like Davina Cooper [41], provide a microcosm in which the foundations of society are both maintained and contested on a frequent basis. Everyday practices matter not only in producing particular public goals but also as a means to socialize and engage individuals within democratic society. We developed the term “everyday democracy,” drawing upon that perspective as well as others [42], in the context of precision health as a means to put health care and medical research in their social context. This enables us to better understand not just how health care and medical research impact society, but also how the role of health care and medical research in society may be used to enable precision health through everyday forms of democracy within health practices. There is also growing evidence that equity within the provision of social services, more generally, is essential to maintain a stable democracy [43].

## Results

### Health Inequity for Marginalized Migrants and Democratic Weaknesses

The Precision Health and Everyday Democracy (PHED) project has been funded from January 1, 2019, for 3 years, but with a 1-year extension owing to COVID-19 travel restrictions (it will run until December 2022). It has so far included a workshop at the University of Texas Medical Branch (Galveston) in April 2019, an international conference cohosted by Lund and Malmö Universities in October 2019, a large 14,3 MEUR application to the EU Horizon 2020 for a project on migrant health care during Spring 2020, and a commission in fall/autumn 2020 with oral and written submissions on the future of health care after COVID-19.

The project is grounded in an empirical focus initially developed through prior study of how marginalized groups are often excluded from health care, specifically, marginalized migrants living in the Skåne region of Sweden.

Earlier research through a research platform, in which several of our network participants are involved (MILSA), has already shown several results regarding the health and well-being of marginalized migrants. A research project within the MILSA platform studied marginalized migrants during 2015 and 2016, who were participating in the establishment process. The investigation showed that migrants self-reported good health, as well as a strong belief that they were able to influence their own health and low use of medications. The most common health problems were mental ill health, allergies, high blood pressure, asthma, diabetes, headaches, stomach pain, muscle pain, and deficient eyesight [3]. The lifestyle factors that could be seen as risk factors for this group of migrants were overweight, obesity, smoking, and physical inactivity. Almost 60% of the respondents have experienced serious threats of violence during the last 12 months preceding their arrival in Sweden, and one-fifth had been exposed to physical violence. The migrants had low confidence in the health care system and the interpreters in Sweden [3]. We have also seen that crowded living increased the odds for recently arrived migrants having mental ill health, but after adjustments were made for stability of housing conditions, the odds decreased [4]. We have also investigated migrants’ experiences of health care services after arrival in Sweden, and both qualitative and quantitative data revealed that migrants had difficulty obtaining the care they sought and experienced dissatisfaction regarding health care [10]. The reasons mentioned for not being able to get health care when needed were cost, long wait times, and language difficulties. Some mentioned having been denied care owing to asylum status and being lost within the referral system and care among different health care settings regarding, for example, diabetes [10]. As mentioned, recently arrived migrants had health and social challenges, and it could be discussed what kind of support and help health professionals need concerning this work within the health care system. Dzur lifts the importance of democratic professionalism and mentions that task sharing within different sites of professional authority could have a core element of democracy [44]. Task sharing could encourage capabilities, interests, and norms of behavior needed for a functioning democracy [44]. This could be a relevant way of introducing democratic perspectives within health care and therefore could support and help health professionals in their engagement for recently arrived migrants.

### Scoping Review

A scoping review was conducted by Mangrio and Sjögren Forss [45] with the aim to compile research about the experiences that refugees worldwide have with the health care system in their respective host countries. The scoping review concluded that communication and information are important factors regarding the experiences that refugees have with health care. For example, in a Canadian study by Chen et al [46], some participants felt insufficient or impersonal communication. In a study by Shannon et al from the United States [47], many participants did not feel comfortable starting a conversation about their war trauma, but would most likely have responded if the doctor initiated the discussion. Two-thirds of the participants were never asked by a doctor about the political conflict in their country or how this may have affected them. In addition, more information needs to be provided about the participants’ health care rights as asylum seekers [48], about their disease [49], and about the delivery room experience, for example, pain medication, why prenatal visits are important, use of interpretation services at the hospital, and what they can expect from the hospital staff [50].

In a Scottish study by O`Donnell et al [51], the results showed that asylum seekers were supposed to receive written information from the health board telling them how to register with a general practitioner, but some did not get this information. Redman et al [52] showed that only nine out of the 30 informants had received information about the free National Health Service and that they wished for even more information about this service. In the Swedish study by Wångdahl et al [53], the results showed that a considerable portion of the informants felt that they received little health care information during the examination and that the quality of communication was low. At least 30% of the informants did not understand what they were being told. Refugees with inadequate health literacy had felt to a lower extent that they received enough health care information and more commonly experienced not receiving any help with health problems.

Regarding the understanding of language during health care visits, Asgary et al [54] mentioned that informants pointed out that there was a lack of interpretation and that they experienced difficulties finding interpreters and were having problems communicating with health professionals. The findings from the report by Bhatia et al [55] confirm those from the report by Asgary et al [54], as the informants in this study also experienced language barrier difficulties and difficulties in obtaining a translator. It was also mentioned that sometimes appointments with doctors had to be rescheduled because no translator showed up. Only those refugees who were accompanied by a relative, friend, or refugee agency staff member could undergo a trouble-free registration process. Cheng et al [56] found that it was difficult for refugees to make appointments because of low proficiency in the English language and that because of the language problem, they preferred verbal reminders over written reminders.

Regarding satisfaction with the health care service among migrants, a Canadian study by Donnelly et al [57] mentioned that many of the participants believed that their health care provider did not spend enough time with them. Consequently, they felt disappointed, and there was distrust in the health care system. In an American study by Asgary et al [54], the authors could see that experiences varied among the participants regarding health care in the United States and that asylum seekers had fear of deportation, detention, and loss of legal status.

## Discussion

### Approach

Marginalized migrants represent an extreme case by which to illustrate health inequity and its wider social and economic consequences. The social exclusions identified in the MILSA project go beyond a concern for the affected individuals since they evidence a degree of segregation in Swedish society, where a high portion of the population, as well as information on their health, risks falling outside the national model for health promotion in the country. The enhancement of precision health requires that such exclusions are ameliorated, meaning increasing access, as well as participation and engagement from a diverse society within Swedish health care and medical research. There is a need to better understand the environmental and genomics data from the full population, including marginalized migrants. Calls for coproduction in health policy and medical research mean not only engaging otherwise often excluded populations, but also considering where they require a focus on sustainability. The ill health of marginalized migrants concerns not just those vulnerable individuals, but has to be seen as a threat to the health security of the broader society. That point has, of course, been well demonstrated during the COVID-19 pandemic, where societies unable to protect their most vulnerable residents, whether granted legal status or not, have had great challenges in controlling the spread of the virus.

Drawing on coproduction, PHED’s transdisciplinary approach means building links across not only academic disciplines, but also professional fields. PHED is engaged therefore with both health care practitioners, such as health clinics, as well as civil society groups (eg, refugee rights organizations) and pharmaceutical firms. While such partners are likely to sometimes have conflicting interests, we consider identifying and working through these tensions as essential to the success of the project as they are part of the structure including and excluding different populations. Pharmaceutical firms that are for profit are easily vilified, and while it is important not to be naïve of their interests, it is necessary to first compare diverse positions and highlight tensions so we can move closer to producing shared knowledge that benefits all sides.

Our consortium therefore seeks to address an urgent challenge to society, namely the requirement to better understand how health practitioners and policy makers can better interact with an increasingly diverse population, as well as to remedy long-standing health inequalities that follow wider societal cleavages and forms of marginalization. To achieve that goal, the proposed multinational and transdisciplinary consortium is intended to internationalize the development of (1) precision health care tools that are sensitive to the needs of a varied population; (2) political science research to identify the social exclusions that undermine precision health care and explore the options for ensuring medical practitioners are better equipped to listen and acknowledge variation among the individuals they treat, using democratic theories; and (3) educational modules (including policy guidance) associated with these research topics. In the long-term perspective, our innovative program is likely to increase the equality of precision health care in Sweden, as well as promote the integration and education of new citizens, followed by an improvement in health equity with additional benefits for Sweden and other regions.

### Time Span and Consortium

The project spans 3 years (2019 through 2021) and brings together scholar scientists and students from five different institutions, the pharmaceutical industry, nonprofit agencies, and three nations. To meet important societal challenges, Lund (coordinator) and Malmö Universities in Sweden will establish an international research and educational consortium characterized by collaborations across the borders of medical, social, political, and biological disciplines. Seed finance has been provided by the Swedish Foundation for International Cooperation in Research and Higher Education (STINT–Stiftelsen för internationalisering av högre utbildning och forskning) to establish the international consortium’s research and education. Within the existing finance, we will not collect new data, and all data collection thus far has been via prior projects that have received ethical approval. Within the consortium, we are targeting a series of core and applied research funds in Sweden and internationally to develop a range of projects that fit within the broad remit of PHED and engage health care and medical research practitioners.

### Planned Activities

We are planning to collaborate with researchers from Brazil and the United States through research, workshops, and conferences, and through this collaboration, we plan to strengthen existing relationships and establish new relations that can benefit Sweden’s internationalization of education, research, and outreach. Brazil and the United States were chosen because of earlier collaborations between Lund University and researchers in these locations. The project is intended to be an open-ended partnership, seeking new collaborators across disciplinary divides to support the transdisciplinary approach essential to the project. We also aim to establish and identify precision health strategies as well as anchor equal access to quality health care for migrants. We will also strive to create educational models for students in methods relevant for precision health care as well as within everyday democracy. The whole project aims to provide junior colleagues (doctoral students, postdoctoral researchers, and junior faculty members) with a dynamic international environment that offers knowledge, new contacts and cultural experiences, leadership training, and career growth. The highly interdisciplinary consortium is held together by its central focus on “precision health.” The concept of “everyday democracy” allows us collectively to enhance ongoing discussions over patient and public participation in health care and medical research, and to develop new insights into the banal practices through which participation can be enabled in a broader societal context.

### Work in Progress

We have work currently ongoing and already completed in this research project. During autumn 2019, we arranged a workshop with the theme precision health and everyday democracy. We invited junior and senior research colleagues from the United States, Canada, India, and Europe and had different topics on the theme. We finalized the whole workshop with presentations from the health care sector and identified relevant challenges for us as researchers to focus our upcoming research on. We ended the whole consortium with a brainstorming meeting around future applications and cowriting of scientific papers. Collaboration between researchers in India, France, and Denmark and the PHED team started from this workshop and ended with an application for Horizon 2020 called PRePARe, which stands for PRogramme for marginalized migrants to Prevent Antimicrobial Resistant infections. It is an innovative and transdisciplinary project for enhanced clinical management and prevention of resistant bacterial infections. This application includes development of an app, which we suggest could enhance the health care access and information that needs to be between asylum seekers and health care professionals. Through this app development, we suggest that the contact and communication between asylum seekers and health care professionals could be improved. We as a research group took the lead in this app, and a total of eight countries were a part of this app. During the last 2 years, we have also been writing scientific papers on the management and prevention of resistant bacterial infection, on COVID-19 regarding the migrant situation health wise [58] and socially, and on migrants and the need for data security when increasing information and health care tasks are being conducted online. During fall 2020, we are having a commission online, which will be for an international audience, and the focus will be on health care access for marginalized migrants, with some focus on the recent pandemic and how this vulnerable group was and is affected. This commission will result in an open-access published report. This and other events within PHED always provide an opportunity for new junior or senior scholars to join the network.

### Conclusions

We are aware of the diversity among worldwide populations, as well as in our collaborating countries Brazil, the United States, and Sweden. We are confident that we can meet the challenges associated with different racial/ethnic groups physically, mentally, and socially. However, we need to identify tools to enable us to both prevent and treat a wide spectrum of health-related outcomes, and better understand how they are linked to social as well as environmental issues. We also need to identify and investigate health care barriers in order for the issues to be dealt with. Our international research team aims and strives toward finding tools and educational models that work to improve these mentioned areas of research. In political science, recent studies have established that democracy promotion can greatly benefit population health and well-being [59-61]. The PHED project goes further by approaching this relationship from the other way round, understanding health equity as not only a normative good but also a contributing factor to a stable and democratic society.

## Appendix

Multimedia Appendix 1 Reviewers' comments confirming approval of the project funding.

## Abbreviations

PHED: Precision Health and Everyday Democracy
