# Supplementary material for: The Precision Health and Everyday Democracy (PHED) Project: Protocol for a Transdisciplinary Collaboration on Health Equity and the Role of Health in Society
Source: JMIR Res Protoc. 2020 Nov 30;9(11):e17324. doi: 10.2196/17324 (PMC7735904; doi:10.2196/17324)
Supplement: Multimedia Appendix 1 [file resprot_v9i11e17324_app1.pdf]

STINT
Stiftelsen för internationalisering av
högre utbildning och forskning
The Swedish Foundation for International
Cooperation in Research and Higher Education

2018-06-26
Dnr: SG2018-7506

Carol Nilsson
Lunds universitet Inst f experimentell medicinsk vet
BMC 113
Sölvegatan 19
22184 LUND

Beträffande ansökan inom Programmet Strategic Grants

Du har ansökt om medel inom programmet för Strategic Grants hos Stiftelsen för internationalisering av högre utbildning och forskning, STINT.

Härmed meddelas att STINT beslutat att bevilja ditt projekt

Beslutsmotiveringen lyder som följer:

Strong commitment to strategic internationalization that is teamed with cross-disciplinary approached from two different Swedish institutions. Proposal is extremely comprehensive although the eventual outcomes of the large planned events will depend on the ability of the project managers to successfully combine the various elements.
Interesting project, but the internationalization benefits seem more implicit than explicit. There certain seems to be some strategic advantage to the international collaboration.
The merger of experimental medicine and pharmacology with larger societal issues is interesting and has great potential to deliver more precise medical assistance to patients in under-served communities.
Offsetting the risk of the project's attempt to cover several disciplines in three countries is the fact that truncating some of the ambitious events would not jeopardize the entire projects; i.e., not every element of the proposed application is necessary for positive results from this endeavor.
There is a bit of high risk here in that the hope is for the funding to lead to future research investments.
The budget is clear. The proportion of budget going to airfare and accommodations is very large but understandable given the project goals of engaging researchers (including new scholars and students) into the events. The project seems reasonable, though I wonder how feasible/functional it is to convene a 50-person workshop. Would a smaller more focused effort be better to kick-off the network?

Har du några frågor är du välkommen att kontakta STINT.

Med vänliga hälsningar

Agneta Granlund

Mäster Samuelsgatan 60, 9 tr.
P.O. Box 3523, SE-103 69 Stockholm, Sweden
Phone +46 8 671 19 90, Fax +46 8 671 19 99
